# Simultaneous microwave digestion for total arsenic and inorganic arsenic in local shrimp and prawn commodities of Brunei Darussalam for regulatory and safety monitoring

**DOI:** 10.1016/j.heliyon.2024.e32224

**Published:** 2024-05-31

**Authors:** M.H. Md Taib, LH Lim

**Affiliations:** a Chemical Sciences, Faculty of Science, Universiti Brunei Darussalam, Tungku Link Road, Bandar Seri Begawan, BE1410, Brunei Darussalam; b Department of Scientific Services, Ministry of Health, Commonwealth Drive, Menteri Besar Road, Bandar Seri Begawan, BB3910, Brunei Darussalam

**Keywords:** Speciation analysis, Prawn, Shrimp, Seafood, Inorganic arsenic, Total arsenic, Regulatory limits

## Abstract

The data gap in food safety regulations have created misinformation leading to the rejection of commodities for trade. The evidence presented is the local regulation of arsenic in sea produce which is based on total arsenic, tAs, instead of toxic inorganic arsenic, iAs. Furthermore, tAs data in animal origin seafood has been widely proven to be dominated by the non-toxic Arsenobetaine, AsB. Therefore, if arsenic regulatory limit was set based only on tAs without reference to iAs data, seafood products might be wrongfully rejected for trade because of non-compliance to tAs limit. We provided analysis of tAs and iAs of 14 local prawn and shrimp commodities from three shrimp/prawn sector namely aquaculture (n = 3), capture (n = 5) and processed (n = 6) using effective extraction, as well as, a fit-for-purpose analytical method for iAs. A HVG-AAS method was developed and validated for iAs with LoQ of 1.6 ppb, analytical range of 0–6 ppb, repeatability RSDr of 0.5–3.1 %, coefficient of determination R^2^ of 0.9975, and percentage recovery of 90.9 %, while an existing method using ICP-MS was used to verify the tAs. Based on the AOAC Official Method 999.10 2005 with minor adjustments, seafood samples were digested with concentrated nitric acid and hydrogen peroxide under pressure in a closed vessel heated by a microwave digester. An additional step for iAs determination was necessary to ensure compatibility in HVG-AAS analysis. Further subdivision of the aquaculture and capture samples was done by dividing them into 3 fractions, namely head, flesh and peel. Comparison of tAs in all the three fractions indicated that for aquaculture sector, the highest tAs were found in the flesh (2nd highest in % weight) whereas for the capture sector, the highest amount of tAs correlated with the highest % weight of the fraction. On regulatory aspects, speciation analysis on the iAs indicated samples with quantifiable iAs value were in-compliance despite tAs were initially found to be higher than the national limits. Risk assessment of iAs indicated there were no risk for human daily intake based on the BDML_0.5_ value of 3.0 μg/kg b.w per day for an average 70 kg man. All findings concluded the need for doing arsenic speciation analysis of iAs along with tAs for routine monitoring of prawn/shrimp samples and to revise the local limits from tAs to iAs particularly for seafood commodities.

## 1 Introduction

In general, the aquatic ecosystem is one of the vast providers of natural resources for human in terms of food supply and trade revenue giving rise to the development of fishery industry for the marine and freshwater organism, which can provide sustenance and livelihood for the people. Food consisting of aquatic animals, collectively known as seafood, covers beyond the apparent term of ‘sea’ to include freshwater species [1], are functional foods rich in proteins and nutrients that provide health benefits for the consumers. Continuous demand for seafood spurred the growth of the fisheries industry in three main sectors of capture, aquaculture and processing. It is usually driven by higher demand from increasing population, urban expansion, higher standard of living, rising global trade and growing preferences towards seafood protein. As reported in 2014, Asia as a region singlehandedly contributed to the majority of the global fish production with 70.8 % which was led by China (37.5 %) followed by Southeast Asia (18.3 %) and South Asia (9.3 %) [2].

In the context of global seafood trade, the aspects of safety and regulations always go hand in hand whereby routine testing in seafood sample is carried out as part of surveillance monitoring to ensure compliance to regulatory limits and trading standards set by national authorities as well as to avoid health risk during human consumption. Rejection or recall of consignments or supplies can occur due to disease outbreaks or non-compliance to the given regulatory limits. The regulatory process is carried out to ensure that the products are safe and nutritious for the customer [3] where this process is also applied to the monitoring of arsenic level in seafood products.

Worldwide regulations on arsenic in seafood typically categorised as fish, fish products, aquatic animals were compiled in Table S1, Supplementary Material, and the values were used to check the compliance of the arsenic content of samples to each of these maximum limits. With the quantitative determination of both total arsenic (tAs) and inorganic arsenic (iAs) for all the samples selected in this research, it can be directly compared for compliance or non-compliance based on the set threshold values.

Total arsenic (tAs) concentrations may be high in certain seafood but several researches [[3], [4], [5], [6], [7], [8], [9]] had shown that for majority of the species, it indicated that about 85 % to >90 % of the total arsenic found in the edible parts of the marine animals is organic arsenic, namely Arsenobetaine (AsB), which is relatively non-toxic to humans. Hence, emphasising the importance of arsenic speciation analysis of the selected arsenic species that are toxicologically relevant to human consumption.

Other literatures, which focused on arsenic speciation analysis and utilising strong acid digestion for tAs and mild extraction for arsenic species such as using methanol/water mixtures or alkaline solution, are able to retain most of the arsenicals present including iAs and organic arsenic (oAs) [1,10,11]. However, with the quantification of iAs in mind, it would be more beneficial to develop a sample extraction method that can be optimized for both tAs and iAs and at the same time with minimal steps. This will enable the simultaneous monitoring of both tAs and iAs for the purpose of comparison and regulatory compliance study. In order to safeguard the seafood industry and facilitate trade, where most global regulatory limits are based on iAs, while our national limits are still relying solely on tAs, there is an urgent need for the testing laboratory to carry out the analysis of both tAs and iAs to prevent wrongfully rejection of seafood products for trade.

Therefore, the main aim of the research is the establishment of monitoring data for the toxic iAs in local prawn/shrimp commodities and the objectives are as follows.

● Development and validation of a rapid and effective method for the simultaneous extraction of tAs and the toxicologically-relevant iAs (speciation analysis of arsenic) in local prawn/shrimp commodities
● Assessment of arsenic content in different sectors of prawn and shrimp in Brunei Darussalam
● Investigation and correlation of contributing factors to the resulting arsenic content in the samples
● Justification for the need to carry out actual iAs determination instead of relying on results based on calculation using conversion of tAs value to iAs
● Increase in awareness of the different toxicity of arsenic species among the relevant agencies and stakeholders based on its chemical nature and oxidation state in order to recommend any changes needed for the legislation

## 2 Materials and methods

### 2.1 Sampling and sub-sampling

Samples were purchased from several local retail supply ranging from supermarket, retail outlet, wet market and even at consumer fair event as detailed in Table S2, Supplementary Materials. Once collected, samples were kept frozen at −20 °C for immediate storage especially for fresh or raw type in order to maintain its integrity, if blending was not able to be done immediately. When it was ready for extraction, each sample was thawed to workable condition before blended until homogenised and it was then kept frozen pending further extraction steps.

**Fig. 1:**
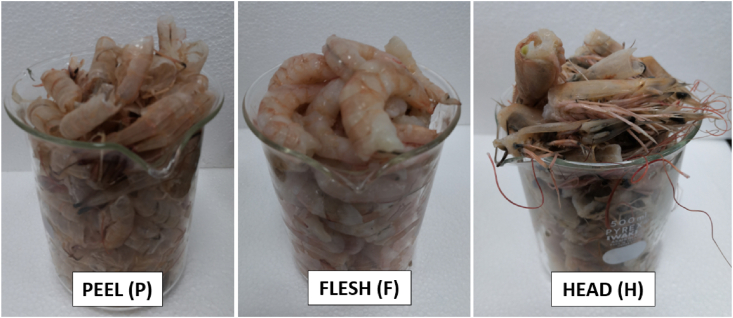
Division of raw prawn and shrimp into 3 fractions: Peel (P), Flesh (F) and Head (H).

Raw shrimp or prawn could be marketed in several configurations such as Head-on Shell-on (HOSO), Shell-on (SO), Peeled Tail-On (PTO), Peel Undeveined (PUD), Peeled and Deveined (P&D), Peel Deveined and Tail-on (PDTO), Butterfly Tail-On (BTTY-TO) and Farmed, Shell-On (FMO). With this in mind, samples of raw commodity i.e. those from the aquaculture and capture sectors underwent additional sub-sampling steps before blending where they were further divided into three fractions: Peel (P), Flesh (F) and Head (H) as shown in Table S3, Supplementary Materials, with the resulting sub-samples as illustrated in Fig. 1. The prawn or shrimp sample was roughly cut or detached along the head and flesh connection line. The head fraction contains the antenna, claw if applicable, content and legs attached. The other half contains the flesh and peel (exoskeleton). The peel was removed manually by hand to include legs and tails attached while separating the flesh into another part. Each fraction was roughly weighed to estimate the proportion from the total sample weight for statistical consideration. Each fraction was extracted separately in order to investigate the variation in tAs and iAs content while the mean values of the three fractions would indicate contribution of the arsenic content from the whole raw shrimp or prawn.

### 2.2 Sample extraction

Samples were blended and homogenised fresh or as purchased using a food processor or a blender. Processed products like crackers or dumplings, remained uncooked and allowed to thaw if required before proceeding to extraction step. No additional cooking steps are implemented for all the samples collected. Sample preparation procedure was adopted from the Association of Official Analytical Chemists (AOAC) Official Method 999.10 2005 with minor adjustments. Briefly, 0.2–0.5 g of homogenised sample was weighed into a digestion vessel and fortified with a known concentration of As if required. A 7 mL aliquot of concentrated nitric acid (HNO_3_) and 1 mL of 30 % hydrogen peroxide (H_2_0_2_) were added and the mixture was left to stand for 5–10 min. Any excessive fuming was allowed to subside before proceeding. The vessel was secured with a screw cap and digested using a microwave digester (Anton Paar or Milestone) where the initial digestion steps were the same for both tAs and iAs analysis but additional steps were required for iAs analysis to ensure compatibility before running in Hydride Vapour Generation Atomic Absorption Spectrometer (HVG-AAS) system as prescribed by the instrument manufacturer [12]. Once digestion was completed, the sample was allowed to cool down to room temperature. This sample digestion is the crucial step particularly for the iAs where any organic arsenicals e.g Arsenobetaine (AsB) and Arsenocholine (AsC) are converted into trimethylarsine oxide (TMAO) and dimethylarsinic acid (DMA) when heated with the nitric acid while DMA is a stable organic compound below 200 °C. Analysis was done in triplicate for each sample including the Certified Reference Material (CRM), spiking samples and process blanks to ensure repeatability check.

The extract solution was transferred to a 50 mL volumetric flask where the cap and vessel were washed several times and the flask was filled to the mark with ultrapure water. The sample extract solution was stored in a 50 mL centrifuge tube for further analysis steps of either tAs or iAs.

For tAs analysis, a sample aliquot of 10 mL was passed through a 0.45 μm syringe filter before analysis using Inductively Coupled Plasma Mass Spectrometry (ICPMS) Agilent 7700x. For iAs analysis, the following steps were adopted based on the *Hydride Vapour Generation Method* (AD-0067) as recommended by Shimadzu [12]: 10 mL of the sample extract was pipetted into a clean beaker for mild heating where the remaining nitric acid was evaporated to dryness using a hot plate ensuring no excessive bubbling. The dried extract was reconstituted with 10 mL of 1 M hydrochloric acid (HCl), to make it compatible for HVG analysis. Another 1 mL of concentrated HCl was added along with an equal volume of 20 % potassium iodide (KI) to allow for reduction of any remaining As(V) into As(III) for better sensitivity [12]. The solution was mixed well and allowed to stand for 30 min. The sample was introduced into the HVG-AAS (Shimadzu HVG-1 and AA-7000) with a continuous flow of sodium borohydride solution (0.4 % NaBH_4_) and 5 M hydrochloric acid which generates the volatile arsines. The signal generated from the AAS would be the sum of arsenite, As(III), and arsenate, As(V), or iAs as whole in the final reading.

### 2.3 Reagents, standards and solutions

Sodium borohydride (NaBH_4_), sodium hydroxide (NaOH), potassium iodide (KI), hydrogen peroxide (H_2_O_2_) 30 %, hydrochloric acid (HCl) 37 % and nitric acid (HNO_3_) 65 % were all analytical grades or equivalent from Merck, Germany. Ultrapure water (UPW) was generated by reverse osmosis, electrodeionisation, ultraviolet (UV) and finally filtered by a 0.22 mm filter to produce water with resistivity of 18 MΩ using the Milli-Q system from Millipore, USA. The Arsenic hollow cathode lamp for AAS was purchase from Photron. Purified gases for ICP-MS (argon and helium) and HVG-AAS (argon and acetylene) were supplied by reputable local vendors.

For ICP-MS, tuning solution at concentration of 10 mg/L As (part no. 5185–5959), internal Standard Mix 100 mg/L (part no. 5188–6525), PA Tuning 1 (part 5188–6524), PA Tuning 2 (part no. 5188–6524) and 1 % Triton X-100 were diluted accordingly for analysis.

For both ICP-MS and HVG-AAS analyses, solid arsenic (III) trioxide with purity ≥99.0 % from Sigma-Aldrich was used for arsenic standard preparation and spiking recovery. Preparation and usage of As(V) standard was not necessary as the final signal reading of the AAS was based on As(III) due to the reduction of any remaining As(V) into As(III) after the addition of equal volumes of HCl and KI. A stock solution of 1000 mg/L of arsenic was prepared by dissolving 0.132 g of arsenic trioxide in 100 mL of 20 % w/v NaOH and then diluted to the 500 mL mark with 1 M HCl. For further dilution of all the working standards, gravimetric dilution using analytical balance was preferred compared to volume dilution using micropipettes.

### 2.4 Quality control

For quality control and traceability purpose, a Certified Reference Material (CRM) was used: BCR-627 Tuna Fish Tissue, which was obtained from the European Commission-Joint Research Centre, Institute for Reference Materials and Measurements (IRMM), Belgium. The certificate of analysis of the CRM provides certified values for AsB (3.90 mg/kg), DMA (0.15 mg/kg) and tAs (4.8 mg/kg) and hence it is applicable for the determination of tAs in ICP-MS and to check for absence of signal during iAs analysis. Additionally, another CRM was used in the ICP-MS analysis i.e NIST 1640a Natural Water which contains tAs concentration of 8.075 parts per billion or ppb.

The CRM BCR-627 does not provide certified iAs value and hence recovery study for iAs using HVG-AAS was carried out by spiking the actual samples that were tested during the same run to account for any pre-existing iAs.

To demonstrate the suitability of both the analytical methods, blank check samples were included in the sample batch run preferably using processed blank i.e. those undergoing the same extraction procedure but without the actual sample. The processed blank value is particularly important for HVG-AAS analysis as it was used as the ‘zero absorbance’ value.

### 2.5 Instrumental analysis

Three main instruments were used for this project namely Microwave Digestion System, ICP-MS and HVG-AAS. The microwave digestion system was used for sample extraction while the ICP-MS and HVG-AAS were for quantification of tAs and iAs, respectively.

All samples were digested using either an Anton Paar Multiwave PRO or a Milestone Start D Microwave Digestion System. The following microwave programme was used: 15 min @1000 Watt 180 °C, 5 min @ 1000 Watt 180 °C.

For the purpose of iAs determination, a Shimadzu AAS system was used (AA-7000 connected to HVG-1). The following is the summary for the main analytical conditions for the HVG-AAS used; wavelength 193.7 nm, lamp current 20 mA, slit width 0.7 nm, background correction of Deuterium lamp, HVG Quartz Cell Heating system of Air-Acetylene flame with acetylene flow rate of 1.8 L min^−1^ and burner height of 16 mm. While tAs determination was done using ICP-MS based on the following instrumental operating conditions; 15 L min^−1^ for plasma gas flow, 0–1.0 L min^−1^ for auxiliary gas flow, 3–5 mL min^−1^ for 3 gases flow i.e. hydrogen gas, helium gas and 3rd cell gas, 26.0–28.0 MHz for plasma frequency, *m*/*z* 75 (^75^As) for monitored signal and 0.30 s for IntegTime/Mass.

### 2.6 Statistical analyses of tAs and iAs in samples

Data analyses were performed using the Microsoft Office Excel 2016 one-way analysis of variance (ANOVA) at 5 % significant level of probability i.e. p-value of <0.05 was used to estimate the significance of difference between the mean concentrations of tAs in the different fractions of the raw commodities of prawn and shrimp namely Peel (P), Flesh (F) and Head (H). The results were expressed as the means ± standard deviation (SD) where each sample was run in triplicates.

The ‘tolerable intake’ is typically used to describe the safe levels of intake for arsenic in food. The Panel on Contaminants in Food Chain (CONTAM) and European Food Safety Authority (EFSA) suggested a range of values of benchmark dose lower confidence limit (BDML) for As, including 0.3–8 μg kg-^1^ of As per kg of body weight per day in case of cancers of lung, skin, bladder and skin lesions [13]. Based on the National Recommended Servings for protein-rich foods [14], the serving for prawn is 2–3 servings per day with 120 g per serving which is equivalent to a daily intake of 240 g–360 g. Considering a typical size of a raw prawn/shrimp of 35–40 g each, 240–360 g would consist of 6–9 whole prawn or shrimp on average.

## 3 Results and discussions

The study highlighted the results for both the method verification for ICP-MS analysis on tAs and the method validation for HVG-AAS analysis for iAs (Sub-Chapter 3.1 and 3.2 respectively). The validated methods were then used for the analysis of the locally available prawn or shrimp commodities and their related products as shown in Sub-Chapter 3.3. While in Sub-Chapter 3.4, the raw commodities which were divided into three different fractions were analysed and the results enabled for comparative study particularly in the distribution of tAs content inside the peel, flesh and head proportions to justify their significance in terms of targeted sampling during sample preparation. Then with the availability of both tAs and iAs data for each sample, regulatory assessment based on the ratio of iAs/tAs was discussed in Sub-Chapter 3.5 in order to check for the presence of the toxic iAs against the tAs as well as comparison with some selected worldwide regulatory limits for arsenic. Lastly, Sub-Chapter 3.6 discussed the safety aspect of the samples for human consumption, where estimated dietary intake of iAs was used as the risk assessment approach to check if the iAs determined in the samples had a significant human health risk based on the benchmark dose lower confidence limit (BMDL) on the recommended daily servings.

### 3.1 Method verification for the analysis of total arsenic (tAs) by ICP-MS

Method verification on the determination of tAs of the prawn/shrimp samples was based on a wet weight basis using ICP-MS was carried out using the standard certified reference material (BCR-627). This determined tAs concentration was then used as the starting point or baseline as screening for the need of further analysis to be carried out i.e the analysis of iAs. Linear range of up to 100 ppb with excellent correlation coefficient of 0.999 was obtained for the ICP-MS analysis.

Limit of Detection (LoD) and Limit of Quantification (LoQ) were calculated by 3.3 times standard deviation (SD) of response divided by Slope and 10 times standard deviation (SD) of response divided by Slope respectively. Standard deviation (SD) of the y-intercepts of the linear regression was used while the slope was estimated from the calibration curve of the analyte. LoD and LoQ of the tAs analysis were calculated to be 1.3 ppb and 4.0 ppb, respectively, allowing for the determination of tAs lower than the regulatory limit of 1 ppm. Table 1 shows a comparison of LoD, LoQ, %RSD and percentage recovery values of this study with those of other literature values. Evidently all the values calculated are comparable in performance irrespective of the use of different instruments and methodology.

**Table 1:** Comparison of LoD, LoQ and Recovery of tAs in this study with those in other literatures.

Reference	Instrument	LoD/ppb	LoQ/ppb	%RSD	Recovery	Matrix
This study	ICP-MS	1.3	4.0	0.90–2.88	71.0–101.6	Shrimp and Prawn Commodities
[5]	ICP-MS	1 2	2 10	NA NA	NA NA	Moist Food Dry Food
[1]	ICP-MS	NA	NA	NA	92	Shrimp wild-caught
[15]	HG-AAS	0.17	0.58	7.5	97.5	Fish
[16]	HG-AAS	0.17	0.57	NA	94–105	Rice products (Solid and liquid)

Recommended range of recovery for tAs based on AOAC SMPR 2012.007 Standard Method Performance Requirements for Heavy Metals in a Variety of Foods and Beverages [17] are 60–115 % for low concentration range of ppb level with repeatability RSD of ≤15 % and LoQ of ≤10 ppb for foods. Based on the recommendation, all the parameters determined in the tAs analysis are considered as satisfactory and hence they are verified as fit for the purpose for this study. Comparison to other studies also illustrated close agreement in terms of the LoD, LoQ, %RSD and percentage recovery obtained.

### 3.2 Method validation for the analysis of inorganic arsenic by HVG-AAS

Similar approach was done for calculating the LoD and LoQ as previously described for tAs where there were calculated to be 0.54 ppb and 1.65 ppb, respectively allowing for the determination of iAs lower than the regulatory limit of 0.5 ppm for iAs from China (Table S1, Supplementary Materials). Sensitivity of the method is the amount of signal per unit concentration of analyte which is also equal to the slope of the calibration curve i.e. 0.0379 while the highest concentration value was 6 ppb for applied calibration standards solutions. No arsenic was detected in the chemicals used in the sample blank with the absorbance value of 0.0150 while the lowest concentration calibration solution was 2 ppb. Based on the method performance requirement of AOAC SMPR 2015.006 Standard Method Performance Requirements (SMPR) for Quantitation of Arsenic Species in Selected Foods and Beverages [18], the minimum acceptance criteria for iAs analysis were compared to. Since quantification of iAs is the priority of the study, LoQ and % recovery of this study were also compared with those of other literatures as summarised in Table 2.

**Table 2:** Comparison of LoQ and Recovery of iAs, As(III) and As(V) found in this study with those in other literatures.

Reference	Instrument	LoD/ppb	LoQ/ppb	Recovery/ppb	Matrix
This study	HVG-AAS	iAs: 0.54 ppb, as sum of As(III) and As(V)	iAs: 1.65 ppb, as sum of As(III) and As(V)	iAs: 90.9 %	Shrimp and Prawn Commodities
[19]	HPLC-ICP-DRC-QMS	As(III): 2.9 ppb	As(III): 9.6 ppb As(V): 2.9 ppb	As(III): 70 % As(V): 135.5 %	Rice
[20]	LC-ICP-MS/MS	Not applicable	As(III): 30 ppb As(V): 26 ppb	Not applicable	Seafood
[16]	HG-AAS	As(III): 1.6 ppb iAs: 4.4 ppb	As(III): 5.4 ppb iAs: 15 ppb	As(III): 92–104 %^a^ iAs: 91–110 %	Rice products
[21]	HPLC-ICP-MS	As(III): 0.06 ppb As(V):0.22 ppb	As(III): 0.19 ppb As(V): 0.65 ppb	iAs: 85.1 %	Drinking water
[22]	LC-ICP-MS	As(III): 1 ppb As(V): 2.4 ppb	As(III): 3.3 ppb As(V): 8.0 ppb	iAs: 100–106 %	Seafood
[23]	High Resolution ICP-MS	Not Applicable	iAs: 30 ppb as sum of As(III) and As(V)	iAs: 101.4 %	Fish, shellfish and seaweed
[11]	IEC/ICP-MS	Not Applicable	As(III): 20 ppb As(V): 20 ppb	Not applicable	Seafood

a Recovery of As(III) in milk powder of 35 % were excluded. The low recovery of As(III) could be due to oxidation of part of the As(III) to As(V) during the extraction.

Other authors reported their LoQ either separately as individual As(III) and As(V) or as combined As(III) and As(V) as shown in Table 2, at concentration range of 0.19 ppb–30 ppb depending on their methods of analyses. The LoQ value of 1.65 ppb for this study was better compared to one study [23] with LoQ of 30 ppb when iAs was analysed in High Resolution (HR) ICP-MS indicating the selectivity advantage of HVG-AAS particularly for iAs. In terms of percentage recovery data, the value of 90.9 % for local prawn was comparable with the recovery values of iAs obtained by the following authors [23,22] of 100–106 % with the similar sample matrix of seafood category.

Table 3 shows a summary of the figures of merit for the HVG-AAS analysis. Although IC or HPLC with ICP-MS would be the preferred analytical technique under the AOAC SMPR 2015.006 for arsenic speciation analysis, HVG-AAS provided a fit for the purpose method that provide quantitation for the sum of iAs in one type of shellfish sample i.e. prawn/shrimp based on the validation data used.

**Table 3:** Summary of validation parameters for HVG-AAS analysis run [18].

Parameter	Range	Minimum acceptance criteria	Actual value obtained
Analytical working range	Depends on ML	10 ppb–1 ppm	0–6 ppb
Linearity	–	0.99 ≤ R^2^ ≤ 1	**0.9975**
Limit of quantitation (LOQ)	Better than ML	<10 ppb	**1.65 ppb**
Recovery (based on spiking sample)	10–100 ppb	60–115 %	NA
	**≥100 ppb – 10 ppm**	**80**–**110 %**	**90.9 %**
Repeatability (RSD_r_)	≤10–50 ppb	≤20 %	–
	**≥50**–**300 ppb**	**≤13 %**	**0.5 % - 3.1 %**
	≥300 ppb–1 ppm	≤12 %	–

Notes: ML: Maximum Limits.

Analytical working range was from 0 to 6 ppb considering it was an HVG-AAS run where it typically utilises a narrower linear working range than an ICPMS run with the range of 0–100 ppb. This in turn requires samples to be diluted to within the working range. This narrow range was comparable to the optimized working range of 0.5–5 ppb by Nugraha et al., 2017 [15], also using HVG-AAS as the analytical instrument. Good linearity was achieved for iAs in this study with R^2^ of better than 0.99. Recovery of spiked samples was found to be in acceptable range of 80–110 % for recovery range of ≥100 ppb–10 ppm. Lastly the triplicates run of every analysis batch generated a good repeatability RSD_r_ value of 0.5%–3.1 % i.e within the recommended range of ≤13 % for concentration values of ≥50–300 ppb [18].

Based on the figures of merit discussed and comparison to other literatures, the HVG-AAS method was validated and hence it is suitable to be used to carry out the speciation analysis of iAs in this study where the method was proven fit for routine testing.

### 3.3 Analysis of local prawns/shrimps commodities and related products

One of the main purposes of this study is to determine the proportion of iAs to tAs where the results of the tAs and iAs are summarised in Table 4. In order to generate a numerical value of % of iAs/tAs ratio for samples with iAs concentration below the LoQ of <0.0016 ppm, the actual value of the LoQ were used i.e. 1.6 ppb or 0.0016 ppm, to calculate the ratio while the LoD was 0.54 ppb This allows the calculation of all the samples with unquantifiable amount of iAs with an exception to C001 (Giant Freshwater Prawn) where both tAs and iAs were below their LoQ hence the resulting % ratio calculated would be 40 % which is an overestimation and not a good representation of the result. On that note, the mean concentration of C001 for the capture sector was also not calculated.

**Table 4:** Summary of analytical results for tAs and iAs in this study.

SAMPLE ID	SAMPLE TYPE	Mean conc ± SD tAs/ppm	Mean conc ± SD iAs/ppm	% of iAs/tAs
A001	White Shrimp 1	1.094 ± 0.114	<0.0016^b^	0.15 %
A002	White Shrimp 2	0.948 ± 0.148	<0.0016^b^	0.17 %
A003	Blue Shrimp	1.720 ± 0.283	<0.0016^b^	0.09 %
**Mean of A**	**AQUACULTURE**	**1.254 ± 0.335**	<0.0016^b^	**0.14 %**
C001	Giant Freshwater Prawn	<0.004	<0.0016^b^	NA^a^
C002	Yellow Shrimp	6.592 ± 0.438	<0.0016^b^	0.02 %
C003	Sea Shrimp	0.404 ± 0.074	<0.0016^b^	0.40 %
C004	Flower Shrimp	0.603 ± 0.156	<0.0016^b^	0.27 %
C005	Tiger Prawn	0.681 ± 0.173	<0.0016^b^	0.23 %
**Mean of C**	**CAPTURE**	**2.070 ± 2.613**	<0.0016^b^	**0.23 %**
P001	Dried acetes	23.084 ± 0.534	0.16 ± 0.02	0.68 %
P002	Dried shrimp	2.393 ± 0.074	0.04 ± 0.02	1.67 %
P003	Shrimp paste	4.605 ± 0.082	0.14 ± 0.04	3.04 %
P004	Shrimp crackers (uncooked)	0.153 ± 0.007	<0.0016^b^	1.05 %
P005	Shrimp dumplings (uncooked)	0.175 ± 0.013	<0.0016^b^	0.91 %
P006	Shrimp shumai (uncooked)	0.481 ± 0.019	<0.0016^b^	0.33 %
**Mean of P**	**PROCESSED**	**5.149 ± 8.176**	**0.112 ± 0.052**	**1.28 %**

Underlined Red Font means tAs concentration that exceeded the National Maximum Limits (MLs) of 1 mg/kg.

a NA: Not Applicable since both tAs and iAs were below the LoQ hence the ratio calculated would be overestimated.

b For iAs below LoQ (0.0016 ppm), iAs is assumed to be 0.0016 ppm to allow for the calculation of a numerical ratio value.

The calculation of % iAs/tAs ratio is based on percentage of the concentration of sum of iAs divided by the concentration of tAs, as an accurate indication for the proportion of the toxic iAs when compared to the tAs. Percentage proportion of iAs/tAs in this study indicated variation across the samples and hence it is not possible to generate any trend for a generalized conversion factor. Similar findings were also reported by various authors showing large variation of the iAs/tAs ratio as summarised in Table S5, Supplementary Materials. Furthermore, according to EFSA [24], the food category under “Fish and other seafood” also presented a particular issue when attempting to generate the iAs values simply by using a conversion factor from tAs. Evidence from literatures showed that there is no consistent relationship between tAs and the iAs in seafood samples as shown in Table S5, Supplementary Materials. One of the reasons is that the relative proportion of iAs tend to decrease as the tAs content increases while the ratio may vary depending on the seafood type [24,25]. Based on the calculated ratio in Table S5, Supplementary Materials. for the comparison with other literature values, % of iAs/tAs were seen to exhibit big variations of 0–22 % even among similar sample matrix and there were no observable trends of consistent ratio being found in an attempt to consider a general conversion factor.

**Fig. 2:**
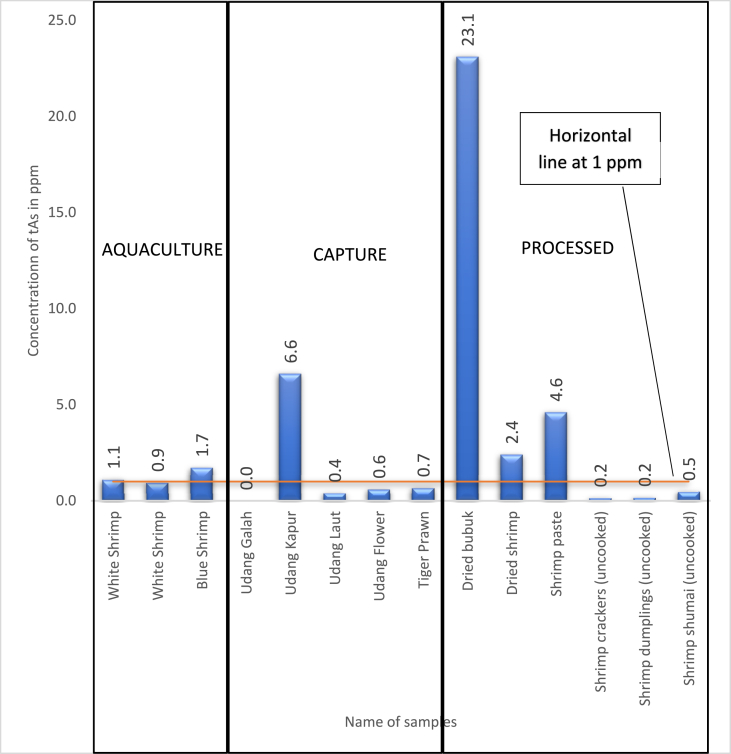
The mean concentration of total arsenic (tAs) in all study samples with comparison to national maximum limits (tAs 1 ppm).

Based on Fig. 2 on the mean tAs value for all the 14 samples, 6 samples namely White Shrimp 1 (A001), Blue Shrimp (A003), Yellow Shrimp (C002), Dried Acetes (P001), Dried Shrimp (P002) and Shrimp Paste (P003) were found to contain tAs of more than 1 ppm i.e the national permissible limit. Only 1 sample i.e Giant Freshwater Prawn (C001) from the capture sector, was not detected of any tAs value (LoD of 1.3 ppb and LoQ of 4.0 ppb). The 6 samples mentioned above would eventually be considered as a non-compliance with regards to the national maximum limits of tAs at 1 ppm and could be rejected for trade where enforcement action might also be taken. However, with the understanding of As toxicity and carrying out further speciation analysis on the toxic iAs along with tAs determination, it revealed that only 3 samples Dried Acetes (P001), Dried Shrimp (P002) and Shrimp Paste (P003) from the processed sectors had detected a relatively low concentration of iAs while the iAs content for the three raw commodities i.e White Shrimp 1 (A001), Blue Shrimp (A003) and Yellow Shrimp (C002) were found to be below the LoD. The absolute values of iAs for the three processed samples, with a range of 0.04–0.16 ppm, were much lower than the lowest and strictest iAs regulatory limit of China i.e. 0.5 ppm.

With this in mind, more arsenic speciation studies, particularly on iAs, should be carried out on other seafood products for a better representation of the overall food groups. However, this preliminary study on local shrimp/prawn commodities had provided evidence that tAs alone should not be used as the sole deciding factor for arsenic contamination investigation in food. Hence the author supported a similar recommendation as suggested by Amlund et al., 2019 [3] and Matos et al., 2022 [4] for countries which are still using tAs as the threshold limit, that any non-compliance should be further investigated with speciation analysis with a particular emphasis on the iAs content. This recommendation is proposed to be included as a statement or instruction in the regulation whereas the establishment of regulatory limit using iAs instead of tAs should also be implemented based on the regulatory limits set by other countries like Thailand, Malaysia and Singapore [26]. This will ensure that samples are not wrongly rejected by only relying on the tAs value.

Although conversion factors had been applied to calculate iAs from tAs value, for instance the assumption of 5–10 % of tAs in seafood is in the form of iAs by the Joint Group of Experts on the Scientific Aspects of Marine Pollution (GESAMP) back in 1986, so iAs was derived from the value obtained for tAs with the 5–10 % conversion factor. However recent studies have disproved the assumption due to variable values of the iAs/tAs ratio that have been reported across the seafood commodities as indicated in Table S5, Supplementary Materials which does not constitute a fixed conversion factor from tAs. Hence the recommendation is whenever possible, dietary exposure should be based on real data on iAs from laboratory testing rather than using calculation based on conversion factors from tAs results. With relation to this, quantitative analysis of iAs is still crucial to be done along with the determination of tAs and whenever arsenic speciation analysis is required and not simply relying on converting the value based on a designated proportion of tAs value.

For raw commodities (aquaculture and capture) samples, no iAs were detected in any of the samples despite detection of tAs (except for Giant Freshwater Prawn sample). However, three of the processed samples i.e. dried acetes, dried shrimp and shrimp paste were detected of iAs as a minor proportion of the tAs ranging from 0.7 to 3.0 %. Based on this, the sample with the highest iAs value i.e. the dried acetes (0.16 ppm) was used to estimate the risk of dietary exposure in Sub-Chapter 3.6 below.

### 3.4 Comparison between fractions of raw commodities

**Fig. 3:**
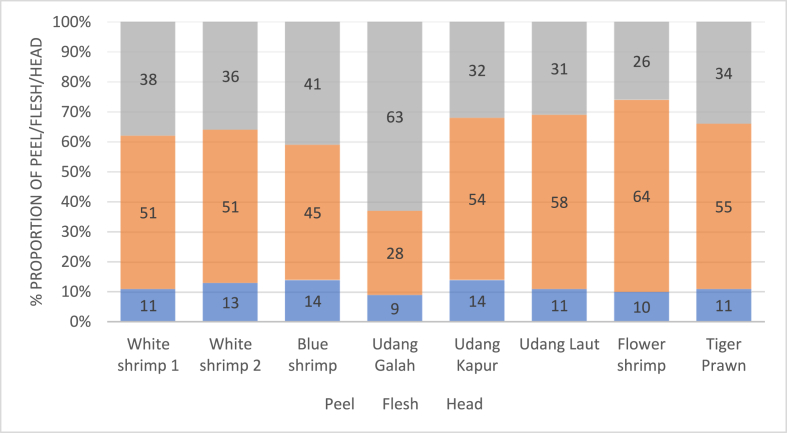
Percentage proportion by gross weights of raw shrimp and prawn commodities for Head, Flesh and Peel Fractions.

**Fig. 4:**
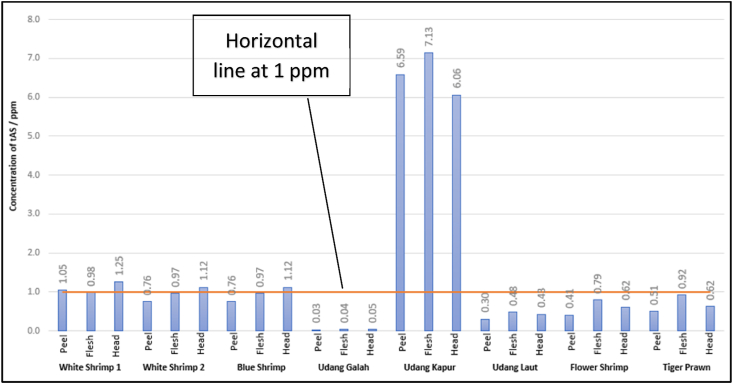
Comparison of tAs concentration in Peel, Flesh, Head Proportion of Shrimp/Prawn Raw Commodities.

**Fig. 5:**
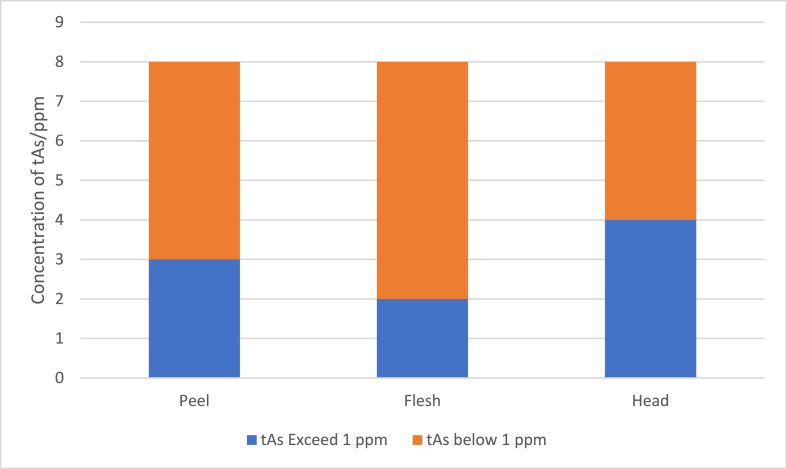
Number of samples of raw commodities based on peel, flesh and head fractions exceeding the tAs of 1 ppm national regulation (non-compliance).

Concentrations of tAs were determined from different fractions (peel, flesh and head) of eight types of raw commodities. The results are illustrated in terms of percentage proportion by gross weights, comparison of tAs concentration and number of samples of raw commodities showing non-compliance to tAs national regulation in Fig. 3, Fig. 4, Fig. 5 respectively while rankings between the different fractions are summarised in Table 5. For the aquaculture commodities, the head portions were observed to have the highest tAs content with all the three species analysed exceeding the national 1 ppm regulatory limit value. Whereas for the capture samples, the flesh proportions were observed to have the highest tAs content with an exception on Giant Freshwater Prawn. With reference to the regulatory limits, all samples were in compliance except for yellow shrimp where all 3 fractions were above 1 ppm.

**Table 5:** Comparison of tAs values between different fractions (Peel, Flesh, Head) of raw commodities.

Category	Sample type	Frac-tion	Estimated % of Fraction Weight/Total Weight)	Mean tAs ± SD (μg g^−1^)	Rank of tAs
AQUACULTURE (Farmed)	White shrimp 1	Peel	11	1.049 ± 0.059	2
		Flesh	51	0.983 ± 0.011	3
		Head	38	1.250 ± 0.160	1
	White shrimp 2	Peel	13	0.758 ± 0.018	3
		Flesh	51	0.965 ± 0.015	2
		Head	36	1.119 ± 0.015	1
	Blue shrimp	Peel	14	0.758 ± 0.018	3
		Flesh	45	0.965 ± 0.015	2
		Head	41	1.119 ± 0.015	1
CAPTURE (Wild-caught)	Giant Freshwater Prawn	Peel	9	0.034 ± 0.001	3
		Flesh	28	0.036 ± 0.002	2
		Head	63	0.045 ± 0.002	1
	Yellow Shrimp (Sea)	Peel	14	6.585 ± 0.740	2
		Flesh	54	7.132 ± 0.106	1
		Head	32	6.059 ± 0.076	3
	Sea Shrimp (Sea)	Peel	11	0.304 ± 0.023	3
		Flesh	58	0.478 ± 0.023	1
		Head	31	0.430 ± 0.003	2
	Flower Shrimp (Sea)	Peel	10	0.406 ± 0.013	3
		Flesh	64	0.788 ± 0.031	1
		Head	26	0.615 ± 0.043	2
	Tiger Prawn (Sea)	Peel	11	0.506 ± 0.022	3
		Flesh	55	0.916 ± 0.004	1
		Head	34	0.622 ± 0.012	2

Higher tAs concentration in the head fractions of the aquaculture samples were expected where most of the internal organs are located which would be prone to metal contaminant absorption including arsenic [4]. Significant level of tAs in all fractions also correlated with the use of salt water in the local aquaculture farms [27] where water salinity would contribute to the presence of arsenic. Feed intake could also contribute to the tAs as reported by Matos et al., 2022 [4] hence further study of arsenic content on the feed for aquaculture commodities would be recommended to check for any correlation.

For the aquaculture samples, no direct correlation was observed between the % weight of the different body parts with the tAs level for all the 3 samples, as the flesh, being the highest % weight, only ranked the second highest in terms of tAs level. Matos et al., 2022 [4] highlighted in their findings that in farmed shrimps, the arsenic content was distributed half in the muscle tissue and half in the head or carapace.

Whereas for the captured samples, there is a high correlation between the weight of the body sections with respect to the level of tAs as reported by Matos et al., 2022 [4] where most of the As was found in the edible part of the shrimp i.e. muscle tissue. This was evident from this study data that samples from the capture sector had the highest tAs in the flesh portion i.e. the highest weight fraction. The only exception was for the Giant Freshwater Prawn where its head fraction being the highest % weight contained the highest tAs.

Based on salinity factor for capture sector, samples originated from the sea exhibit a significant tAs content although only one sample, yellow shrimp, showed elevated values of 6–7 times the national threshold limit. When compared with the only fresh water sample of 10.13039/100005130Giant Freshwater Prawn, where the tAs were significantly lower in all fractions hence supporting the contribution of salinity factor for arsenic presence [4].

Based on the results of the one-way ANOVA in Table 6, Tables S6 and S7 Supplementary Materials for comparison between different fractions of the raw commodities with p > 0.05, df = 2, F_stat_ < F_crit_, the analyst accepted the null hypothesis where there is no significant difference between the means of the 3 groups which implied that the means of tAs between the three fraction of Peel, Flesh and Head are all equal.

**Table 6:** tAs value for different fraction of raw commodities (triplicate samples) for ANOVA calculation.

Label	Type of sample	Concentration of tAs (in parts per million, ppm)		
		Peel	Flesh	Head
A001	White shrimp 1	0.97	0.99	1.43
		1.07	0.99	1.27
		1.11	0.97	1.04
A002	White shrimp 2	0.75	0.96	1.10
		0.78	0.98	1.14
		0.74	0.95	1.12
A003	Blue shrimp	1.29	1.97	1.66
		1.44	2.03	1.82
		1.35	2.14	1.77
C001	Giant Freshwater Prawn	0.03	0.03	0.04
		0.03	0.04	0.04
		0.03	0.03	0.05
C002	Yellow Shrimp (Sea)	5.56	7.28	6.03
		6.90	7.10	6.16
		7.29	7.02	5.98
C003	Sea Shrimp (Sea)	0.33	0.50	0.43
		0.30	0.45	0.43
		0.28	0.48	0.43
C004	Flower Shrimp (Sea)	0.40	0.81	0.56
		0.39	0.74	0.67
		0.42	0.81	0.62
C005	Tiger Prawn (Sea)	0.52	0.91	0.62
		0.52	0.92	0.61
		0.48	0.92	0.64

However, observing the incidence of tAs value exceeding the national limits in different fraction of raw commodities, there is a variation in value showing in ascending order are flesh < peel < head where the highest incidences are in the head fractions with 12 samples compared to 8 samples for Peel followed by 6 samples for flesh fraction. Looking at the different types of samples, the aquaculture sector tends to have tAs of flesh < head while capture samples tend to have tAs of head < flesh with an exception to Giant Freshwater Prawn. This observation is consistent with the results reported by Matos et al., 2022 [4] despite no separate investigation was done on the peel. While for the farmed shrimps, the As distributions were predominantly in the Carapace (part of the head, 53 %) and Muscle Tissue (or flesh, 47 %) whereas for wild shrimps, the As distribution were predominantly on the muscle tissue (64 %) and carapace (33 %). Based on this study and other literatures, it might not be sufficient to do sampling only on the edible part i.e. flesh for routine tAs monitoring. The peel and head should also be considered to ensure a better statistical representation of each sample. Despite of the flesh fraction being the main proportion of the commodities with the exception of Giant Freshwater Prawn, it would be expected to contribute the most for arsenic contamination. However, depending on the demand for arsenic analysis, some clients might request specific parts of the crustaceans to be investigated especially if it will be used for a specific purpose for example the head and peel being used for shrimp flavour in processed products or broth. One research strategy utilised by Matos et al., 2022 [4] has conducted pooled sampling instead of individual sampling for better representation of each species type or category.

### 3.5 Proportion of iAs/tAs in different types of prawn/shrimp and comparison with other studies

It was evident that raw commodities of aquaculture and capture had undetectable level of iAs despite generating tAs of over the national MRL of 1 ppm. However, the mean value for processed commodities apart from being the highest for tAs, the proportion of iAs only contributed 2.2 % of the total arsenic quantified. Therefore, despite high value of tAs exceeding the national threshold of 1 ppm, iAs had shown to be very low or negligible value. Table 7, which compares the findings of this study with various other studies, showed that almost all prawn and shrimp samples showed elevated amount of tAs beyond the 1 ppm of the national regulatory limits with an exception to shrimp [1] and tiger prawn [28]. In terms of iAs, all of the listed commodities were only found to have trace levels or non-detected levels of iAs, indicating a negligible proportion of the iAs out of the tAs. These results indicated the need to carry out the analysis of iAs for the assessment of As contamination in a sample. With reference to worldwide regulatory limits of arsenic in seafood-related matrices (Brazil, Brunei, China, Malaysia, Australia, New Zealand, Singapore and Thailand), study data were compared to all values of maximum limits based on available data on tAs and iAs as summarised in Table 8. Several incidences of non-compliance were observed under the national regulatory limit of 1 ppm specifically for tAs. Aquaculture products (n = 2), capture/wild-caught products (n = 1) and processed or value-added products (n = 3) showed values of beyond 1 ppm of tAs.

**Table 7:** Comparison of mean concentration of tAs and iAs (if applicable) in this study with other literatures by sectoral categories (Aquaculture, Capture, Processed).

Categories	Sample type	tAs/ppm	iAs/ppm	Extraction method	Reference
**Aquaculture products**	Shrimp	1.254 ± 0.335	<0.0016	Acid microwave digestion	This study
	Shrimp	0.133 ± 0.011	<0.005	Microwave assisted acid decomposition	[1]
	Tiger Prawn	0.798 ± 0.048	Not Analysed		[28]
	Whiteleg Prawn	**1.051 ± 0.124**	Not Analysed		
	Peneaus Monodon	**2.7 ± 0.5**	Not Analysed		[29]
**Capture/Wild-caught products**	**Wild-caught prawn/shrimp**	**2.07 ± 2.61**	**<0.0016**	**Acid microwave digestion**	**This study**
	Wild-caught shrimp	**10.25 ± 0.37**	<0.005	Microwave assisted acid decomposition	[1]
	Marine shrimp S1	**13.3 ± 3.19**	0.06		[30]
	Marine shrimp S2	**9.22 ± 0.79**	0.06		
	Marine shrimp S4	**8.52 ± 0.49**	0.03		
	Marine shrimp S8 (Alpheus rapacida)	**11.0 ± 1.57**	0.03		
	Marine shrimp S8 (Metapenaeopsis palmensis)	**16.7 ± 1.44**	0.13		
	Marine shrimp S9	**20.8 ± 3.51**	0.10		
	Marine shrimp S11 (Alpheus rapacida)	**27.6 ± 5.98**			
	Marine shrimp S11 (Metapenaeopsis palmensis)	**25.3 ± 2.79**			
	Marine shrimp S12 (Alpheus rapacida)	**17.8 ± 6.09**			
	Marine shrimp S12 (Metapenaeopsis palmensis)	**20.3 ± 3.95**			
**Processed or Value-Added Products**	**Shrimp/Prawn based products**	**5.149 ± 8.176**	**0.112 ± 0.052**	**Acid microwave digestion**	**This study**
	Shrimp paste	**6.16**	Not Analysed		[31]
	Dried shrimp	**4.03**	Not Analysed

**Table 8:** Evaluation of mean tAs and iAs value in this study for compliance with worldwide regulatory limits.

CATEGORIES	SAMPLE TYPE	Mean conc tAs/ppm	tAs<1 ppm^a^	Mean conc iAs/ppm	iAs<0.5 ppm^b^	iAs<1 ppm^c^	iAs<2 ppm^d^
**Aquaculture products**	White Shrimp 1	1.094 ± 0.114	Exceed	<0.0016	Comply	Comply	Comply
	White Shrimp 2	0.948 ± 0.148	Comply	<0.0016	Comply	Comply	Comply
	Blue Shrimp	1.720 ± 0.283	Exceed	<0.0016	Comply	Comply	Comply
**Capture/Wild-caught products**	Udang Galah	<0.004	Comply	<0.0016	Comply	Comply	Comply
	Yellow Shrimp	6.592 ± 0.438	Exceed	<0.0016	Comply	Comply	Comply
	Sea Shrimp	0.404 ± 0.074	Comply	<0.0016	Comply	Comply	Comply
	Flower Shrimp	0.603 ± 0.156	Comply	<0.0016	Comply	Comply	Comply
	Tiger Prawn	0.681 ± 0.173	Comply	<0.0016	Comply	Comply	Comply
**Processed or Value-Added Products**	Dried acetes	23.084 ± 0.534	Exceed	0.157 ± 0.02	Comply	Comply	Comply
	Dried shrimp	2.393 ± 0.074	Exceed	0.04 ± 0.02	Comply	Comply	Comply
	Shrimp paste	4.605 ± 0.082	Exceed	0.14 ± 0.04	Comply	Comply	Comply
	Shrimp crackers (uncooked)	0.153 ± 0.007	Comply	<0.0016	Comply	Comply	Comply
	Shrimp dumplings (uncooked)	0.175 ± 0.013	Comply	<0.0016	Comply	Comply	Comply
	Shrimp shumai (uncooked)	0.481 ± 0.019	Comply	<0.0016	Comply	Comply	Comply

a Brazil (fish), Brunei Darussalam (fish and fish products).

b China (aquatic animals and their products).

c Malaysia (fish and fishery products).

d Australia and New Zealand (Fish and Crustacea), Singapore (Fish and Crustacea), Thailand (Aquatic animal and seafood).

Therefore, relying solely on tAs alone as the regulatory limit, will result in potentially making the products non-compliance for the local markets. However, further investigation using As speciation analysis techniques particularly on the toxic iAs showed that all of the products are in compliance to even the lowest iAs limits i.e. 0.5 ppm of China for aquatic animal and their products.

The regulatory limit for tAs as set by Brazil and Brunei Darussalam for commodities relevant to prawn and shrimp is 1 ppm. The tAs value for the white shrimp 1 might also be considered as borderline case especially with the uncertainty value. In this case, further speciation analysis would help to justify that it does not contain iAs level of over the permissible limit. Many countries have opted for iAs as their national limits instead of tAs and China has the lowest limit of 0.5 ppm [32] whereas 1.0 ppm iAs for Malaysia [26] and 2.0 ppm iAs for Australia, New Zealand, Singapore and Thailand. With iAs commonly used as the MLs across the region, the local testing laboratories should be readily equipped with facilities that are able to carry out arsenic speciation analysis especially when there is intention to export the prawn/shrimp commodities to other countries.

Considering a similar two-tier action which is recommended by the FAO/WHO for fats and oils (CXS 329–2017), if the first tier was to conduct tAs testing despite the iAs level being set as the current regulatory limit (using 0.5 ppm iAs as the lowest ML), then only four out of the 14 samples are in compliant with the recommendation limits. The remaining ten samples would need to further undergo iAs determination as the second tier action in order to check for the actual proportion of iAs present. This showed that iAs analysis would still be required as part of the routine testing because AsB was proven to be the main arsenicals presents in shrimp and prawn [1,5,6,22,[20], [33], [34]] so relying on tAs value alone could potentially resulted in the wrong regulatory action.

### 3.6 Risk assessment based on estimated dietary intake of iAs

Back in 2009, EFSA requested a revision of Provisional Tolerable Weekly Intake, PTWI, of inorganic arsenic from all sources (i.e. 0.0015 mg/kg body weight) and introduced a range of benchmark dose lower confidence limit (BMDL_01_) values of between 0.3 and 8 μg/kg b.w per day [24]. A similar verification was performed by JECFA [35] which proposed that the BMDL_0.5_ value at 3.0 μg/kg b.w per day contributing to a 0.5 % increase in lung cancer rates as determined from epidemiological studies. With the recommended daily servings of 360 g for shrimps for an average adult of 70 kg, the calculation is done as shown below for the highest level of iAs with value of 0.16 ppm on dried acetes sample.

Using concentration of iAs in dried acetes = 0.16 ppm (μg/g)

For 360 g servings, concentration of iAs = 0.16 μg/g x 360 g = 57.6 μg.For an average adult of 70 kg,

Estimated dietary exposure to iAs = 57.6 μg iAs/70 kg b.w per day = 0.82 μg/kg b.w per day.

Estimating dietary exposure for the other 2 processed samples with quantifiable iAs concentration of 0.04 ppm for dried shrimp and 0.14 ppm for shrimp paste, resulted in values of 0.21 μg/kg b.w per day and 0.72 μg/kg b.w per day respectively as shown in Table 9.

**Table 9:** The estimated daily intake in 3 processed samples from this study based on the mean iAs obtained.

BMDL_0.5_ (μg/kg b.w per day)	Estimated Daily Intake EDI (μg/kg b.w per day)		
	Dried Acetes	Dried Shrimp	Shrimp Paste
3.0	0.82	0.21	0.72

Based on the guidelines of BMDL_0.5_ 3.0 μg/kg b.w per day for increased risk of cancer of the lung, skin and bladder as well as skin lesions, the estimated dietary exposure for all three processed samples ranged from 0.21 to 0.82 (μg/kg b.w per day). The highest BMDL_0.5_ value of 0.82 (μg/kg b.w per day) determined in this study is still lower than the BMDL_0.5_ value and hence indicating that there is no risk for human daily intake of iAs via prawn and shrimp commodities where similar results were also reported from other study [10]. In 2014, EFSA also reported that the mean dietary exposure to iAs in food among European adults were also within the range of BMDL_01_ i.e. 0.13–0.56 μg/kg b.w. per day for average consumers and 0.37–1.22 μg/kg b.w. per day for 95th percentile (high level) consumers. It was further concluded that the possibility of a health risk to some consumers cannot be excluded especially for children under the age of 3 years where the effect is estimated to be 2–3 fold higher [24]. Despite the health risk concern, seafood is still not the main contributor of dietary iAs and it is typically consumed in moderation. More concerns should be raised on cereal grains and cereal-based products instead as the leading contributors of iAs in food and in terms of regional context where rice is considered as staple food, were shown to be a more significant exposure of iAs than seafood [24].

## 4 Conclusion

Based on the research project, the generation of arsenic speciation data in one particular type of seafood i.e. prawn or shrimp showed paramount importance because it will not be sufficient to rely on secondary data to recommend changes to the regulation. Regional variation can happen based on the aquatic environment, industry and eating habits hence further emphasising on generating local arsenic speciation data. Apart from being utilised as Trading Standards or Reference for Food Safety Monitoring for enforcement agency and seafood industry regulators, regulatory limits also act as a useful guideline for routine testing laboratory to ensure their analytical performances are able to meet or surpass the regulatory limits being set.

Current worldwide regulations on iAs are still scarce despite iAs is being recognised as a human carcinogen [3]. As shown previously in Table S1 Supplementary Materials, only a handful of countries have established maximum limits for iAs namely Australia, New Zealand, Canada, China and France whereas only Brazil and Brunei Darussalam have opted for tAs value. International Organisations namely FAO/WHO have not established maximum limits of arsenic for prawn/shrimp but they do have values for fish oils commodities based on CXS 193–1995 General Standard for Contaminants and Toxins in Food and Feed.

Amlund et al. (2019) [3] suggested that for seafood in general where iAs usually constitutes a small proportion of the total arsenic, it is more suitable to base future legislation on iAs rather than tAs as in the case for prawn and shrimp commodities. Because if the latter value is set as the limits, seafood that are high in tAs and yet low in iAs are at risk of being wrongfully assess and probably rejected from being marketed or traded. It was also suggested that there is a need for standardisation of methodologies for the determination of iAs in preparation if future regulation established maximum levels for iAs in seafood. Furthermore, gathering occurrence data of iAs in seafood would be vital for risk assessments. In terms of the investigation on the three fractions of the raw commodities, it was shown statistically that the means of tAs for peel, flesh and head fractions were all equal and has no significant difference. Hence sample analysis should be sufficient to be done on any of the fractions or typically on flesh or muscle as the most consumed part however utilising the whole prawn/shrimp consisting of the peel, flesh and head would be a more viable option for a better representation of the results especially when the peel and head were used as natural flavouring agents for processed foods.

## Funding statement

This research was supported and funded by the 10.13039/100009100Universiti Brunei Darussalam, Brunei Darussalam. M. H. Md Taib's study was funded under scholarship by the Public Service Department, Brunei Darussalam and supported by the Department of Scientific Services, 10.13039/100009647Ministry of Health, Brunei Darussalam.

## Data availability statement

Data associated with this study has not been deposited into a publicly available repository and will be made available on request.

## Ethics statement

The study was reviewed and approved by the Joint Research Ethics committee of PAPRSB Institute of Health Science (IHSREC), Universiti Brunei Darussalam and Ministry of Health (MHREC), with the approval number UBD/PAPRSIHSREC/2020/136.

## CRediT authorship contribution statement

**Muhamad Hilmi Md Taib:** Writing – review & editing, Writing – original draft, Validation, Methodology, Formal analysis, Conceptualization. **Lee Hoon Lim:** Writing – review & editing, Writing – original draft, Validation, Supervision, Methodology, Funding acquisition, Formal analysis, Conceptualization.

## Declaration of competing interest

The authors declare the following financial interests/personal relationships which may be considered as potential competing interests:Muhamad Hilmi Md Taib reports administrative support and equipment, drugs, or supplies were provided by 10.13039/100009647Ministry of Health Department of Scientific Services. If there are other authors, they declare that they have no known competing financial interests or personal relationships that could have appeared to influence the work reported in this paper.
