# Supplementary material for: Simultaneous microwave digestion for total arsenic and inorganic arsenic in local shrimp and prawn commodities of Brunei Darussalam for regulatory and safety monitoring
Source: Heliyon. 2024 May 31;10(11):e32224. doi: 10.1016/j.heliyon.2024.e32224 (PMC11180320; doi:10.1016/j.heliyon.2024.e32224)
Supplement: Multimedia component 1 [file mmc1.docx]

**APPENDIX A**

**SUPPLEMENTARY MATERIALS**

HELIYON-D-23-26052R1

Supplementary Table(s) with caption

***Table S1: Selected Worldwide regulatory limits for arsenic on related seafood and their products***

| **Country / Region / Organization** | **Maximum Limit (Year Established or Last Amended)** | | **Seafood Group** | **Reference & National Agenc(ies)** |
| --- | --- | --- | --- | --- |
|  | **Total Arsenic (tAs)** | **Inorganic Arsenic* (iAs)** |  |  |
| Australia and  New Zealand | - | 2 mg/kg (2015) | Fish and Crustacea | [Arsenic (foodstandards.govt.nz)](https://www.foodstandards.govt.nz/consumer/chemicals/arsenic/pages/default.aspx#:~:text=There%20are%20limits%20in%20the%20Code%20for%20inorganic,is%20not%20allowed%20above%20a%20level%20of%202mg%2Fkg.) |
| Brazil | 1 mg/kg (2013) | - | Fish | ANVISA (National Agency for Sanitary Surveillance) Resolution No. 42 (from 29^th^ August 2013) |
| Brunei Darussalam | 1 ppm (2001) | - | Fish and fish products | Public Health (Food) Regulations (1^st^ February 2001) |
| Canada | 3.5 ppm (2014) | - | Fish protein concentrate | [Chem-Canada 2015.pdf (fisheries.go.th)](https://www.fisheries.go.th/quality/analyse/chem/Chem-Canada%202015.pdf) Canada Food Inspection Agency, Fish Products Standards and Methods Manual, Appendix 3 Canadian Guidelines for Chemical Contaminants and Toxins in Fish and Fish Products, (Date modified 2014-08-11) |
| China | - | 0.5 mg/kg (2012) | Aquatic animals and their products | National Food Safety Standard Maximum Levels of Contaminants in Food. Issued by Ministry of Health of the People’s Republic of China (Issued on 13/11/2012) |
|  | - | 1 mg/kg (2012) | Fish and their products |  |
| FAO / WHO | - | 0.1 mg/kg (2017) | Edible fats and oils (including fish oils) | General Standard for Contaminants and Toxins in Food and Feed CXS 193-1995 (Amended 2019) |

***Table S1: Continue***

| **Country / Region / Organization** | **Maximum Limit (Year Established or Last Amended)** | | **Seafood Group** | **Reference & National Agenc(ies)** |
| --- | --- | --- | --- | --- |
|  | **Total Arsenic (tAs)** | **Inorganic Arsenic* (iAs)** |  |  |
| Malaysia |  | 1 mg/kg | Fish and fishery products | Food Regulations 1985 (Updated until Jan 2014) |
| Singapore | - | 2 ppm (2020) | Fish and Crustaceans | Maximum Limits for Marine Biotoxins, Inorganic Arsenic and Methanol in Food-Circular (17 March 2020) |
|  | - | 1 ppm (2020) | Molluscs |  |
| Thailand | - | 2 mg/kg (2003) | Aquatic animal and seafood | Thailand Ministry of Public Health. 2003. Notification of the Ministry of Public Health no. 273 (B.E. 2546). Food residues (second issue). The Government Gazette, Vol. 120, Special Issue 77D, 16 July 2003. Bangkok, Thailand |

***Table S2: Information of study samples consisting of prawn / shrimp commodities and related products***

| **Sample Image and ID Number**  **[C:Capture, A:Aquaculture, P:Processed]** | **Common Name** | **Scientific Name of Prawn/Shrimp** | **Sample Description/ Condition** |
| --- | --- | --- | --- |
| 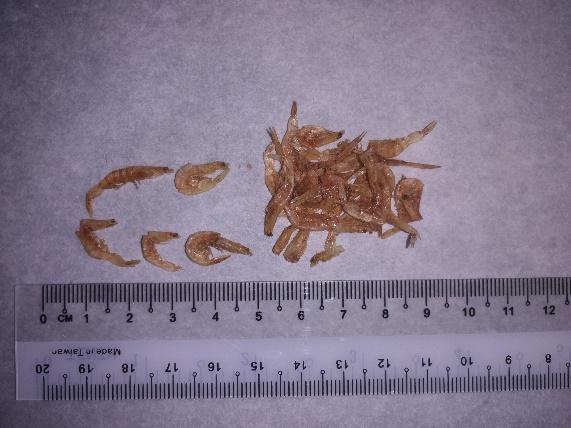  P001/04/21 | Bubuk Kering or Dried Acetes | *Acetes* | Dried,  Room Temp. later Frozen for storage |
| 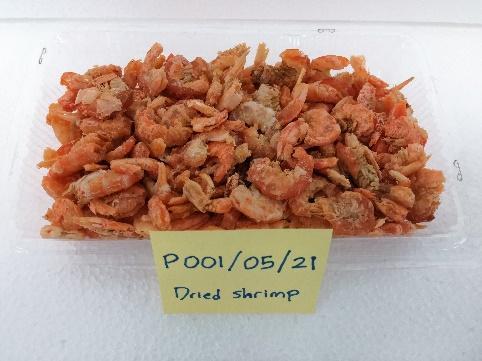  P002/05/21 | Dried Shrimp or ‘Udang Kering’ | Not available | 300 g, Dried, Chilled |
| 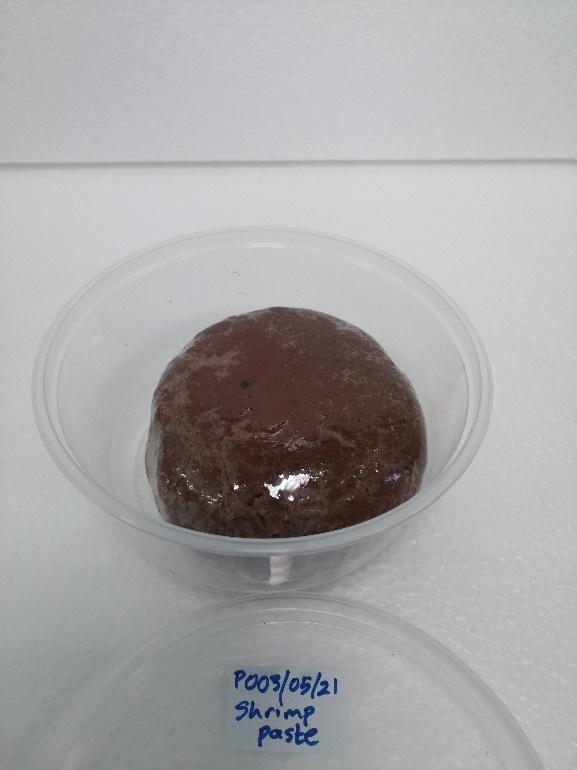  P003/05/21 | Shrimp Paste or ‘Belacan’ | *Acetes* | 450 g,  Chilled  Usually made from fresh Acetes or Bubuk |
| 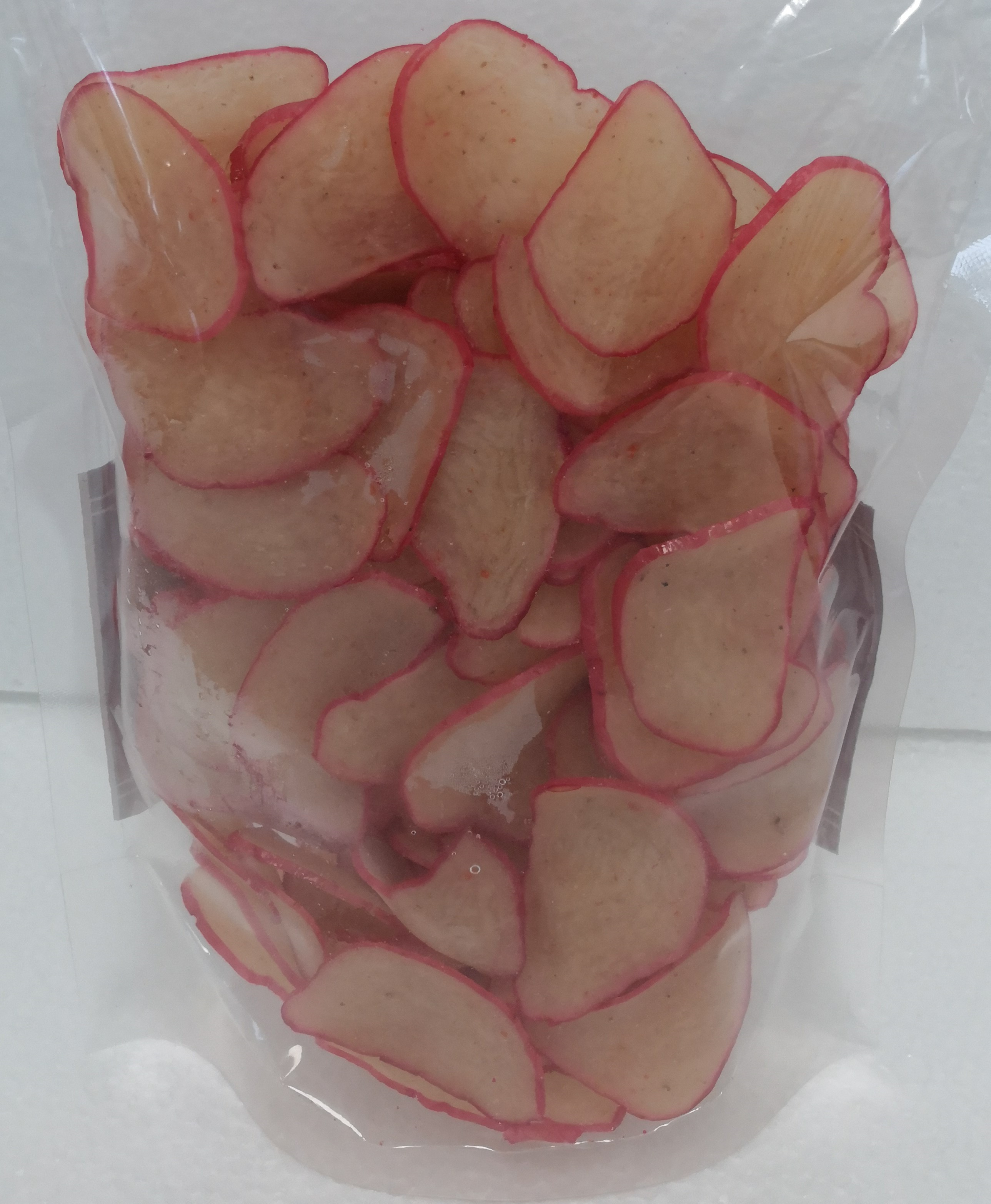  P004/05/21 | Shrimp Crackers or ‘Keropok Udang’ | *Vannemei* or *Rostris* | 250 g, Room Temperature. |

***Table S2: Continue***

| **Sample Image and ID Number**  **[C:Capture, A:Aquaculture, P:Processed]** | **Common Name** | **Scientific Name of Prawn/Shrimp** | **Sample Description/ Condition** |
| --- | --- | --- | --- |
| 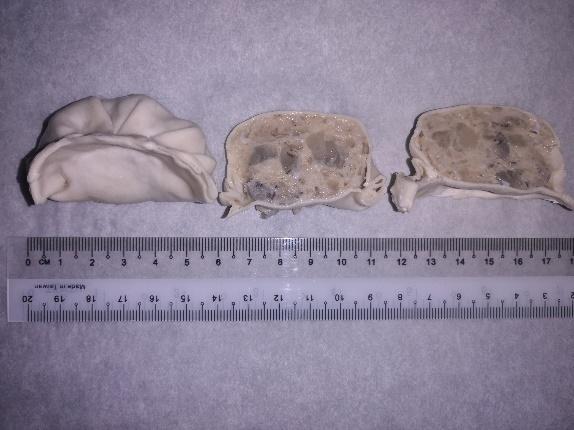  P005/05/21 | Shrimp Dumplings | *Vannemei* or *Rostris* | 15 pcs, Frozen |
| 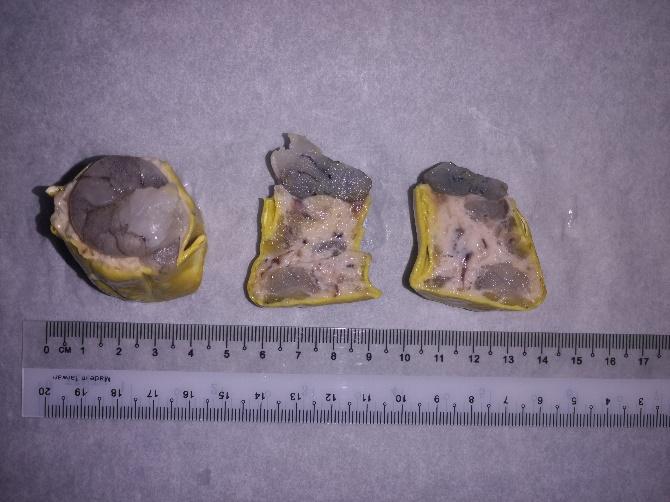  P006/05/21 | Shrimp Shumai | *Vannemei* or *Rostris* | 15 pcs, frozen  Shrimp: Flesh only, no peel and head |
| 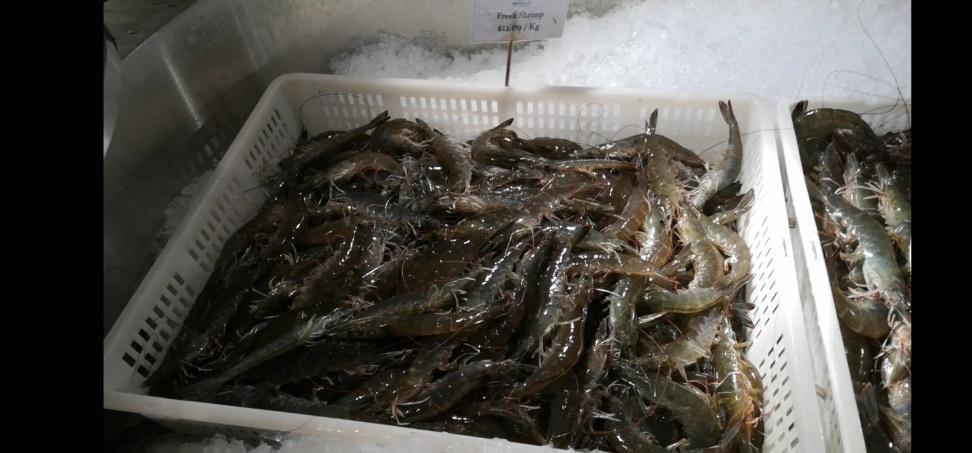  A001/05/21 | White Shrimp | *Vannamei* | 1 kg, Fresh  Origin: Aquaculture Farm |
| 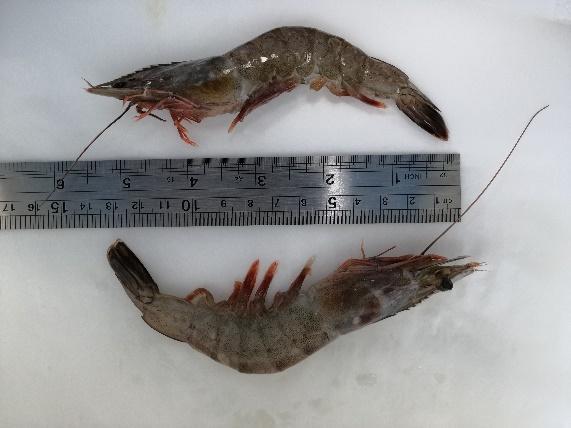  A002/05/22 | White shrimp | *Vannamei* | 800g, Frozen  Origin: Aquaculture Farm |

***Table S2: Continue***

| **Sample Image and ID Number**  **[C:Capture, A:Aquaculture, P:Processed]** | **Common Name** | **Scientific Name of Prawn/Shrimp** | **Sample Description/ Condition** |
| --- | --- | --- | --- |
| 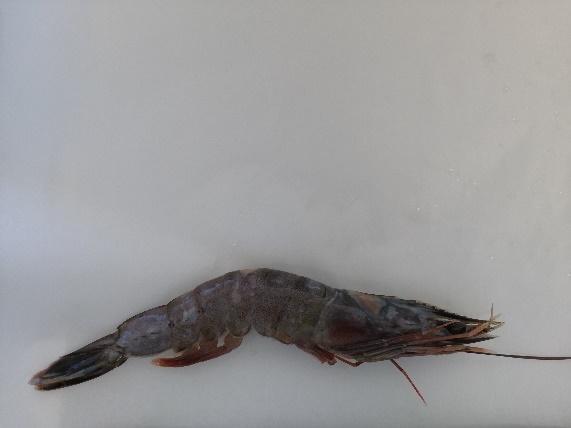  A003/05/22 | Blue shrimp | *Rostris* | 400g x2, Frozen  Origin: Aquaculture Farm |
| 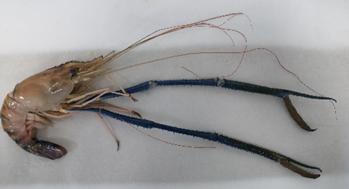  C001/06/21 | Giant Freshwater Prawn or ‘Udang Galah’ | *Macrobrachium rosenbergii* | 1 kg, iced  Origin: River |
| 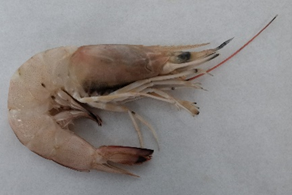  C002/06/21 | Yellow Shrimp or ‘Udang Kapur’ | *Metaenaeus brevicornis* | 1 kg, iced  Origin: Sea |
| 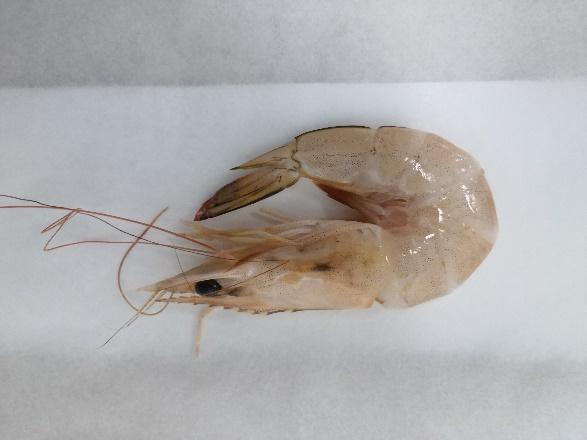  C003/06/21 | Sea Shrimp or ‘Udang Laut’ | *-* | 1 kg, iced  Origin: Sea |

***Table S2: Continue***

| **Sample Image and ID Number**  **[C:Capture, A:Aquaculture, P:Processed]** | **Common Name** | **Scientific Name of Prawn/Shrimp** | **Sample Description/ Condition** |
| --- | --- | --- | --- |
| 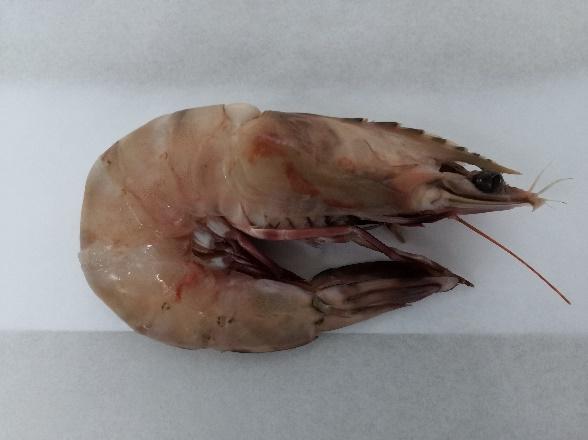  C004/06/21 | Flower Shrimp or ‘Udang Bunga’ | *Penaeus semisulcatus* | 1 kg, iced  Origin: Sea |
| 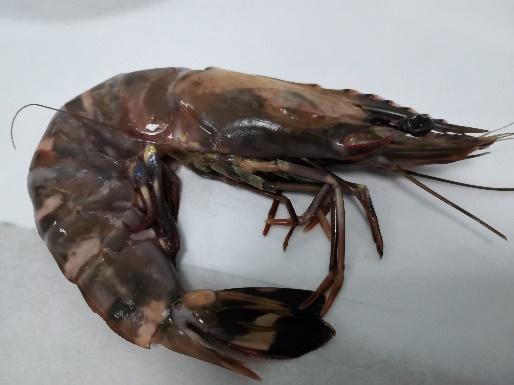  C005/06/21 | Tiger Prawn or ‘Udang Harimau’ | *Penaeus Monodon* | 1 kg, iced  Origin Sea |

***Table S3: Sub-sampling of raw commodities of prawn/shrimp***

| 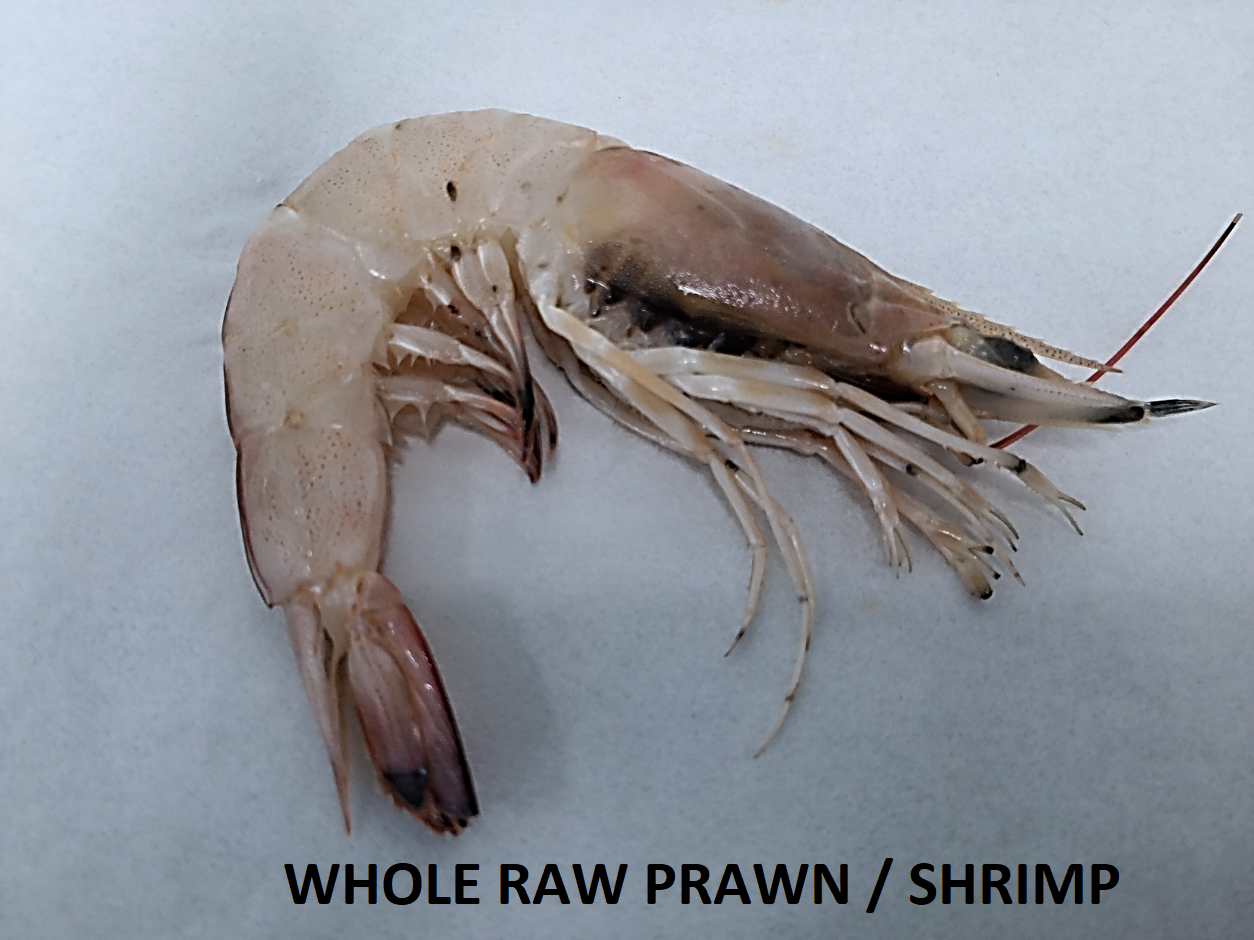 | | |
| --- | --- | --- |
|  | | |
| **Illustration of fraction** | **Label** | **Description** |
| 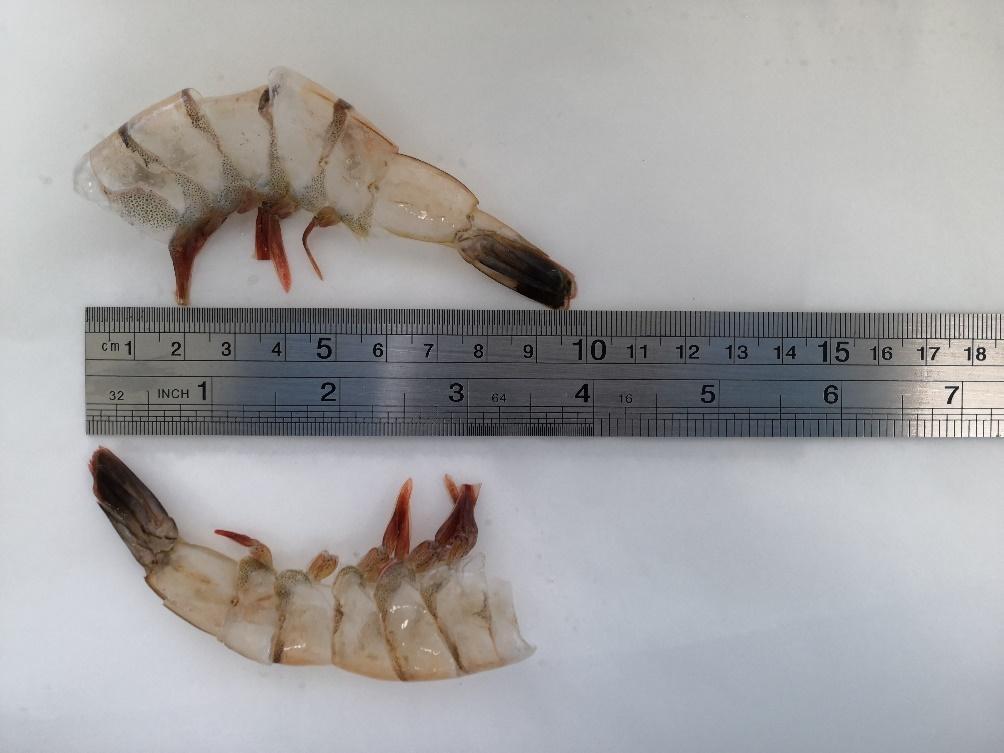 | **P** | PEEL: outer shell / exoskeleton including tail and legs attached |
| 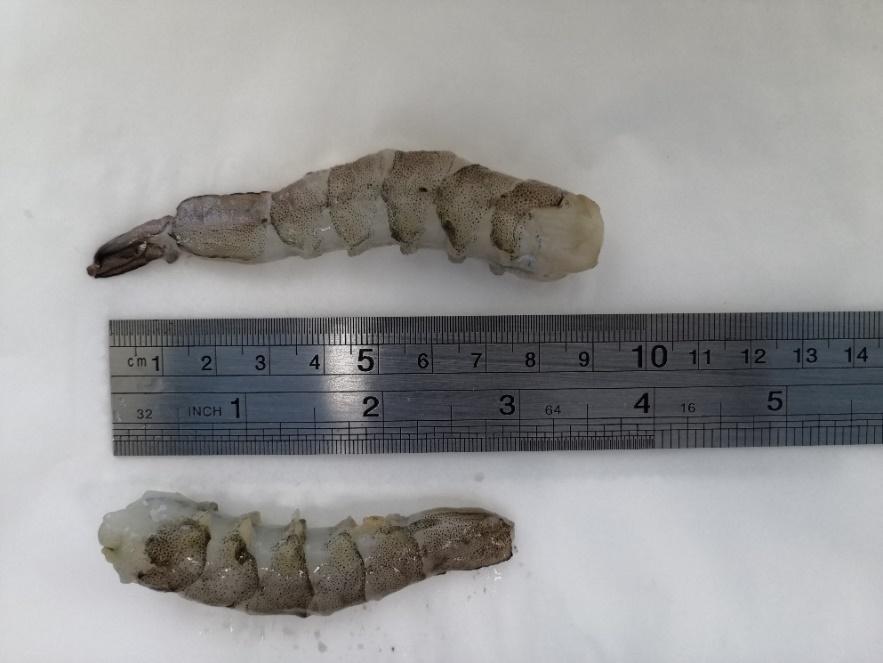 | **F** | FLESH: including “vein” i.e. digestive tract |
| 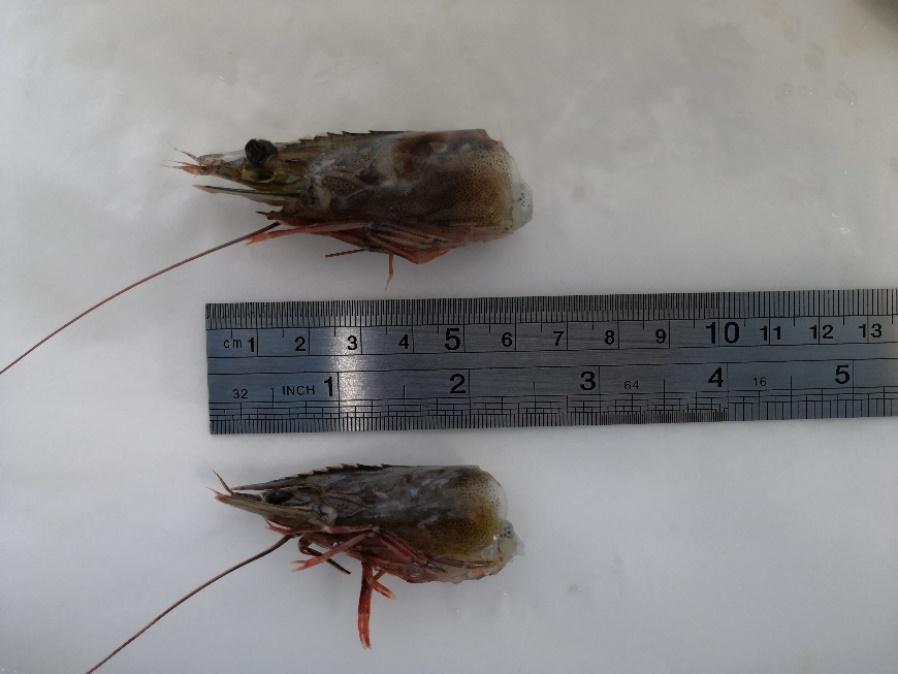 | **H** | HEAD: including carapace, antenna, claw, organ content and legs attached |

***Table S4: Typical absorbance value for calibration standards of iAs determination by HVG-AAS***

| **Concentration of iAs / ppb** | **average absorbance**  **(a)** | **corrected absorbance**  **(a – 0.0150)** |
| --- | --- | --- |
| 0 | 0.0150 | 0 |
| 2.22 | 0.1083 | 0.0933 |
| 4.20 | 0.1827 | 0.1677 |
| 6.26 | 0.2520 | 0.2370 |

***Table S5: Comparison of arsenic species data % of iAs/tAs and/or % of AsB/tAS in different seafoods (concentration in parts per billion, ppb)***

| **Reference** | **Seafood sample type** | **Organic Arsenic (oAs)** | | | **Inorganic Arsenic (iAs)** | | | | **Unknown. Arsenic** | **∑As species detected** | **Total Arsenic (tAs)** | **% of iAs / tAs** | **% of**  **AsB / tAs** |
| --- | --- | --- | --- | --- | --- | --- | --- | --- | --- | --- | --- | --- | --- |
|  |  | **AsB** | **MMA** | **DMA** | **As(III)** | **As(V)** | | **∑iAs** |  |  |  |  |  |
| [18] | White fish | 33.5 | 0.014 | ND | - | | - | ND | 0.1 | 33.6 | 34.3 | 0% | 98% |
|  | Red porgy | 33.2 | 0.010 | ND | - | | - | ND | 0.94 | 34.1 | 33.8 | 0% | 98% |
|  | Hake-1 | 6.58 | ND | ND | - | | - | ND | 0.07 | 6.65 | 6.70 | 0% | 98% |
|  | Hake-2 | 3.2 | 0.012 | 0.13 | - | | - | ND | 0.142 | 3.41 | 3.8 | 0% | 84% |
|  | Forkbeard | 20.3 | ND | 0.24 | - | | - | ND | 4.53 | 25.0 | 27.6 | 0% | 74% |
|  | Sardine | 5.27 | ND | 0.16 | - | | - | ND | ND | 6.0 | 6.88 | 0% | 77% |
|  | Salmon-1 | 1.18 | ND | 0.012 | - | | - | ND | ND | 1.21 | 1.45 | 0% | 81% |
|  | Salmon-2 | 0.86 | ND | 0.03 | - | | - | ND | ND | 0.93 | 1.38 | 0% | 62% |
|  | Tuna-1 | 0.90 | ND | 0.05 | - | | - | ND | 0.05 | 1.08 | 1.41 | 0% | 64% |
|  | Tuna-2 | 1.43 | ND | 0.02 | - | | - | ND | ND | 1.46 | 1.71 | 0% | 84% |
|  | Louvar | 4.15 | ND | 0.04 | - | | - | ND | 0.09 | 4.3 | 4.65 | 0% | 89% |
|  | Swordfish-1 | 4.20 | ND | 0.16 | - | | - | ND | 0.35 | 4.73 | 5.20 | 0% | 81% |
|  | Swordfish-2 | 1.73 | ND | 0.05 | - | | - | ND | 0.89 | 2.68 | 3.00 | 0% | 58% |
|  | Swordfish-3 | 1.96 | ND | 0.05 | - | | - | ND | 0.16 | 2.17 | 2.58 | 0% | 76% |
|  | Prawn-1 | 1.44 | 0.08 | 0.06 | - | | - | 0.06 | 0.01 | 1.66 | 2.0 | 3% | 72% |
|  | Prawn-2 | 2.21 | 0.012 | ND | - | | - | 0.037 | 0.101 | 2.37 | 2.9 | 1% | 76% |
|  | Shrimp | 0.61 | 0.016 | ND | - | | - | 0.033 | 0.052 | 0.70 | 1.0 | 3% | 61% |
|  | Clam-1 | 11.7 | ND | 0.25 | - | | - | 0.35 | 2.58 | 15.4 | 16.8 | 2% | 69% |
|  | Clam-2 | 7.93 | ND | 0.14 | - | | - | 0.20 | 1.93 | 10.21 | 10.5 | 2% | 76% |
|  | Mussel | 8.79 | ND | 0.07 | - | | - | 0.08 | 0.98 | 10.0 | 10.3 | 1% | 85% |
|  | Cockle | 4.01 | 0.13 | ND | - | | - | 0.27 | 1.04 | 5.5 | 7.5 | 4% | 53% |
|  | Oyster | 15.9 | 0.08 | 0.10 | - | | - | 0.10 | 0.75 | 17.1 | 21.7 | 0% | 73% |
| [26] | Shark | 13.9 | <0.012 | <0.006 | - | | - | <0.03 | <0.026 | 13.9 | 14.5 | 0.2%* | 96% |
|  | Shrimp | 2.06 | <0.012 | <0.006 | - | | - | <0.03 | <0.026 | 2.06 | 2.11 | 1.4%* | 98% |
|  | Squid | 1.31 | <0.012 | <0.006 | - | | - | <0.03 | 0.14 | 1.45 | 1.50 | 2.0%* | 90% |
|  | Oyster | 5.00 | <0.012 | 0.70 | - | | - | 0.26 | <0.026 | 6.36 | 6.92 | 4% | 72% |
|  | Scallop | 0.58 | <0.012 | <0.006 | - | | - | <0.03 | 0.15 | 0.73 | 0.74 | 4.1%* | 78% |
| [24] | *Konosirus Punctatus* (fish) | 1.18 | ND | 1.35 | ND | | ND | ND | - | 2.53 | 2.98 | 0% | 40% |
|  | *Chelon haematocheilus* (fish) | 2.02 | ND | ND | ND | | ND | ND | - | 2.02 | 2.23 | 0% | 91% |
|  | *Sebastes schlegelii* (fish) | 3.06 | ND | ND | ND | | ND | ND | - | 3.06 | 3.51 | 0% | 87% |
|  | Crab | 3.87 | 1.47 | ND | ND | | ND | ND | - | 5.34 | 5.98 | 0% | 65% |

**N.D: Not Detected**

***% of iAs/tAs were calculated using iAs of 0.03 ppb when the sum of iAs were <0.03**

***Table S5: Continue***

| **Reference** | **Seafood sample type** | **Organic Arsenic (oAs)** | | | **Inorganic Arsenic (iAs)** | | | | | | | **Unknown. Arsenic** | **∑As species detected** | | **Total Arsenic (tAs)** | | **% of iAs / tAs** | **% of**  **AsB / tAs** |
| --- | --- | --- | --- | --- | --- | --- | --- | --- | --- | --- | --- | --- | --- | --- | --- | --- | --- | --- |
|  |  | **AsB** | **MMA** | **DMA** | **As(III)** | | **As(V)** | | **∑iAs** | | |  |  |  |  |  |  |  |
| [6] | Scallop | - | - | - | - | | | - | | 1.1 | - | | | - | | 12.4 | 9% | - |
|  | Whelks | - | - | - | - | | | - | | 2.2 | - | | | - | | 238 | 1% | - |
|  | Ray | - | - | - | - | | | - | | 0.7 | - | | | - | | 61 | 1% | - |
|  | Dogfish | - | - | - | - | | | - | | 1.4 | - | | | - | | 238 | 1% | - |
|  | Lemon Sole | - | - | - | - | | | - | | 1.7 | - | | | - | | 237 | 1% | - |
|  | Pelagic Fish Group | - | - | - | - | | | - | | 0.32 | - | | | - | | 18.9 | 2% | - |
|  | Drum | - | - | - | - | | | - | | 0.15 | - | | | - | | 8.9 | 2% | - |
|  | Catfish | - | - | - | - | | | - | | 0.14 | - | | | - | | 42.5 | 0% | - |
|  | Corvina | - | - | - | - | | | - | | 0.11 | - | | | - | | 3.5 | 3% | - |
| [25] | Silver bream | 0.0752 | - | - | - | | ND | | - | | | - | 0.0752 | | 0.0900 | | - | 84% |
|  | Silver bream | 0.0880 | - | - | - | | ND | | - | | | - | 0.0880 | | 0.116 | | - | 76% |
|  | Bream | 0.447 | - | - | - | | ND | | - | | | - | 0.447 | | 0.518 | | - | 86% |
|  | Carp | 0.0604 | - | - | - | | ND | | - | | | - | 0.0604 | | 0.0660 | | - | 92% |
|  | Bream | 0.3008 | - | - | - | | 0.0101 | | - | | | - | 0.3109 | | 0.379 | | - | 79% |
|  | Trout | 3.87 | - | - | - | | 0.1337 | | - | | | - | 4.00 | | 4.52 | | - | 86% |
|  | Sturgeon | 5.23 | - | - | - | | 0.0379 | | - | | | - | 5.27 | | 5.932 | | - | 88% |
|  | Trout | 4.16 | - | - | - | | 0.0570 | | - | | | - | 4.22 | | 4.822 | | - | 86% |
| [1] | Shrimp wild-caught | 9.458 | - | 0.0189 | - | | <5 | | - | | | 0.770 | - | | 10.25 | | - | 92% |
|  | Shrimp aquaculture | 0.0104 | - | 0.0029 | - | | <5 | | - | | | 0.120 | - | | 0.133 | | - | **8%** |
|  | Coho salmon wild-caught | 0.165 | - | 0.0353 | - | | <5 | | - | | | 0.160 | - | | 0.354 | | - | 47% |
|  | Coho salmon aquaculture | 0.473 | - | 0.0133 | - | | <5 | | - | | | 0.110 | - | | 0.597 | | - | 79% |
|  | Geoduck clam | 0.491 | - | 0.491 | - | | 0.201 | | - | | | 1.60 | - | | 3.733 | | - | **13%** |
| [5] | Fish products (gratinated, canned, pickled) | 0.779 | 0.253 | 0.098 | - | - | | | 0.062 | | | - | 1.192 | | 1.678 | | 4% | 46% |
|  | Freshwater fish (striped catfish, carp) | 0.013 | 0.023 | ND | - | - | | | 0.008 | | | - | 0.032 | | 0.037 | | 22% | 35% |
|  | Marine fish (including tuna) | 2.393 | 0.040 | 0.229 | - | - | | | 0.010 | | | - | 2.672 | | 2.821 | | 0% | 85% |
|  | Migratory fish (eel, salmon, trout) | 0.413 | 0.112 | 0.049 | - | - | | | 0.021 | | | - | 0.476 | | 0.605 | | 3% | 68% |
|  | Seafood (including shrimp/prawn) | 1.222 | 0.226 | 0.161 | - | - | | | 0.017 | | | - | 1.626 | | 1.717 | | 1% | 71% |
| **N.D: Not Detected** | | | | | | | | | | | | | | | | | | |

***Table S6: Summary of parameters to calculate ANOVA***

| *Groups* | *Count* | *Sum* | *Average* | *Variance* |
| --- | --- | --- | --- | --- |
| mean tAs for Peel | 24 | 33.00 | 1.37 | 4.28 |
| mean tAs for Flesh | 24 | 40.04 | 1.67 | 4.75 |
| mean tAs for Head | 24 | 35.68 | 1.49 | 3.38 |

H_0_: All means are equal

H_A_: Not all means are equal, at least two are different

α=0.05

***Table S7: Values of one-way ANOVA for different fractions of raw commodities***

| *Source of Variation* | *SS* | *df* | *MS* | *F_stat_* | *P-value* | *F_crit_* |
| --- | --- | --- | --- | --- | --- | --- |
| Between Groups | 1.1 | 2 | 0.5 | **0.1** | **0.9** | **3.1** |
| Within Groups | 285.4 | 69 | 4.1 |  |  |  |
| Total | 286.4 | 71 |  |  |  |  |

SS: The sum of squares due to the source

*df*: The degree of freedom in the source

MS: The mean sum of squares due to the source

***Table S8: Typical absorbance value for calibration standards of iAs determination by HVG-AAS***

| **Concentration of iAs / ppb** | **average absorbance**  **(a)** | **corrected absorbance**  **(a – 0.0150)** |
| --- | --- | --- |
| 0 | 0.0150 | 0 |
| 2.22 | 0.1083 | 0.0933 |
| 4.20 | 0.1827 | 0.1677 |
| 6.26 | 0.2520 | 0.2370 |

HELIYON-D-23-26052R1

Supplementary Figure(s) with captions

***Figure S1: Typical Standard Calibration Curve for Inorganic Arsenic (iAs) run in HVG-AAS***
